# The Sport Concussion Assessment Tool (SCAT2) for evaluating civilian mild traumatic brain injury. A pilot normative study

**DOI:** 10.1371/journal.pone.0212541

**Published:** 2019-02-20

**Authors:** Andreea Rădoi, Maria A. Poca, Darío Gándara, Lidia Castro, Mauricio Cevallos, Maria E. Pacios, Juan Sahuquillo

**Affiliations:** 1 Neurotraumatology and Neurosurgery Research Unit (UNINN), Vall d'Hebron Research Institute (VHIR), Barcelona, Spain; 2 Universitat Autònoma de Barcelona, Barcelona, Spain; 3 Department of Neurosurgery, Vall d’Hebron University Hospital, Barcelona, Spain; 4 Neurotraumatology Emergency Unit, Vall d´Hebron University Hospital, Barcelona, Spain; University of Florida, UNITED STATES

## Abstract

Self-report measures, particularly symptom inventories, are critical tools for identifying patients with persistent post-concussion symptoms and their follow-up. Unlike in military or sports-related assessment, in general civilian settings pre-injury levels of concussion-like symptoms are lacking. Normative data are available in adolescent and college populations, but no reference data exist to guide clinical adult explorations. The purpose of this study was to use the second edition of the Sport Concussion Assessment Tool (SCAT2) to profile a cohort of 60 healthy community volunteers who had not sustained a head injury. Participating volunteers underwent MRI scanning and were evaluated with the Hospital Anxiety and Depression Scale (HADS). Participants reported a median of 3 concussion-like symptoms and the 97.5 percentile score was found at 10.5 symptoms, out of a total of 22. The median severity score was 4.9 points, and 28.9 was the upper limit of the reference interval. Only 10 participants (16.7%) did not endorse any symptom. The most frequently endorsed symptom was feeling difficulty in concentrating, with 41.7% of the sample reporting it. Age, sex and general distress, anxiety and depressive symptoms were not associated with concussion-like symptoms. Our data yielded elevated cut-offs scores for both the number of symptoms and the symptom severity. In conclusion, postconcussive-like symptoms are frequent in the general non-concussed adult population and it should be taken into account in any future models developed for screening patients at risk of developing physical, cognitive, and psychological complaints following mild traumatic injury.

## Introduction

Mild traumatic injury (mTBI) and specifically concussion as a result of traffic accidents, assaults, sports, work injuries or deployed military have been acknowledged as a topic of intense public concern in the last decade.[1, 2] Concussion may translate into somatic symptoms—dizziness, nausea, headaches, etc.—and may also affect cognitive and emotional functioning, and in some patients these consequences can be long-lasting.[3] Recent multidisciplinary clinical and research efforts have been addressed at improving mTBI diagnosis, tracking recovery and identifying patients at risk of experiencing persistent post-concussion symptoms (PPCS) such as headaches, dizziness, fatigue, sleep disturbances and/or cognitive problems.

The American Congress of Rehabilitation Medicine defined mTBI in 1993 as any ‘traumatically-induced physiological disruption of brain function manifested at least by loss of consciousness of less than 30 minutes, posttraumatic amnesia (PTA) not greater than 24 h, any alteration of the mental status or transient or non-transient focal neurological deficits’.[4] However, the controversy regarding the operational definition of mTBI diagnosis is ongoing.[5] Some consider the term ‘concussion’ equivalent to mTBI and the term includes mechanically-induced brain dysfunctions at the mild end of the severity spectrum. The discussion around the implications of the use of terms ‘concussion’ and ‘mTBI’ is beyond the scope of this paper, but the reader is referred to the comprehensive review by Sharp and Jenkins.[6] For the sake of simplicity, in this paper concussion refers to any mTBI without any evidence of structural brain damage in the computed tomography (CT) scan. However, there is increasing evidence that up to 20–30% of mTBI patients with normal CT scan show significant brain changes either in structural or functional magnetic resonance imaging (MRI).[7]

Several tools and scales have been designed and used for the clinical assessment of mTBI.[8] All of them include an inventory of self-reported symptoms, frequently applied together with neuropsychological and postural equilibrium tests.[8] Although symptom reporting has become the most used strategy to predict concussion outcome, the symptoms triggered by concussion are notoriously heterogeneous and non-specific. Patients with no TBI history but presenting other conditions (chronic pain, depression, etc.) endorse many concussion-like symptoms and, in some cases, with similar severity.[9, 10] In addition, cohorts described as ‘healthy’ populations also endorse concussion-like symptoms at what could be considered a clinically relevant rate.[11, 12]

One of the most widely used tools in mTBI is the Sport Concussion Assessment Tool (SCAT), a scale proposed by the international Concussion in Sport group as part of a comprehensive concussion screening instrument in sports.[13, 14] The original scale contained 18 symptoms as a measure of an individual’s status following TBI, in addition to other 7 items specifically designed for follow-up visits.[13] In 2009, the International Symposia on Concussion in Sport consensus statement proposed the second version of this tool (SCAT2).[15] The SCAT2 is a brief and easy-to-use tool that puts together self-reported symptoms and objective evaluation of cognitive deficits and post-concussion signs. SCAT2 has various subsections and among them a 22-item self-reported symptom checklist in which each symptom’s severity is rated in a Likert scale from 0 to 6.[15] Additional components include a 2-item physical signs score, the Glasgow Coma Scale, a modified Balance Error Scoring System (M-BESS), a coordination examination and a cognitive Standardized Assessment of Concussion (SAC) that evaluates memory, orientation and concentration. Although several updated editions of the SCAT have been put into use, the symptom checklist has remained unchanged up to the most recent edition, which is SCAT5.[16]

Since 2004, the World Health Organization (WHO) Collaborating Centre Task Force on mTBI has recommended post-concussive symptoms to be assessed in conjunction with psychosocial or injury-related factors (pain, depression, anxiety, posttraumatic stress, litigation status, etc.).[17] In particular, psychological distress has been consistently linked with more severe early symptomatology and with a slower recovery.[17] As such, one hypothesis is that baseline psychological wellbeing can modulate SCAT performance. Putukian et al. have shown that, in college students, depression and anxiety levels were associated with SCAT2 baseline higher severity scores and higher number of symptoms, although not with SAC or balance scores. In this study, athletes endorsing baseline depressive symptoms and/or anxiety reported worse symptom severity and more symptoms in the SCAT2.[18]

Although the SCAT2 was initially designed for sideline examination in sports-related concussion, it has been increasingly used in the clinical setting.[19, 20] The general approach in using the different SCAT versions is to measure the athlete’s post-trauma performance in comparison with a baseline evaluation collected before season.[21] However, in civilian assessment of concussion, a baseline SCAT score is never available and therefore the same approach is useless. The aim of this study was to explore the frequency of concussion-like symptoms and their severity in a healthy civilian population. Our goal was to establish population-based symptoms and severity score thresholds that can be used in future multivariate analysis and supervised-machine learning models of non-sport mTBI studies and, furthermore, to evaluate whether the concussion-like symptoms endorsed by the cohort were influenced by age, sex or anxiety and depressive symptoms. Supervised machine-learning models that screen for patients at risk of developing persistent post-concussion symptoms could increase their accuracy—sensitivity, specificity and predictive value—by identifying the patients with concussion-like symptoms that are common in the healthy population.

## Participants and methods

### Setting and participants

The Traumatology Hospital at the Vall d’Hebron University Hospital is an academic tertiary referral center with a translational research program in TBI and a comprehensive neurorehabilitation facility with expertise in TBI. One of the ongoing research projects is a prospective study on the outcome of mTBI in an adult civilian cohort, in which a supervised-machine learning approach will be used to discriminate patients at high risk of developing persistent post-concussion symptoms. To avoid selecting an arbitrary cut-off outcome based on SCAT2, we carried out a pilot study to understand the baseline characteristics of a civil population and to explore the potential number of symptoms and their severity that could define a clinically relevant threshold.

Between April 2013 and August 2017, next-of-kin or companions of patients admitted to the Neurosurgery Department of our institution—for completing clinical studies or surgery—were invited to enroll in a control group for the mTBI study. The recruitment was made in a general neurosurgical department with a wide variety of diseases that require surgery (hydrocephalus, lumbar or cervical disk surgery, brain tumors, etc.), and no participants were related to TBI patients admitted in our center. Inclusion criteria were to be between 18 and 65 years of age and to proficiently speak Spanish and/or Catalan. Exclusion criteria were: 1) history of TBI, regardless of severity; 2) history of chronic abuse of psychoactive substances or alcohol; 3) known psychiatric or neurologic disorder; 4) chronic systemic disease with known repercussions on the cognitive status by itself or its treatment (cancer, kidney or liver failure, metabolic syndrome, etc.). Participants reported their education status as the highest level completed as well as the number of full-time years of study completed. The following equivalence can be established: 8 years for primary education, 10 years for secondary education, 12 or 13 years for high-school or professional studies, and 1 more year for each full-time undergraduate and postgraduate school-year. In a WHO national survey conducted in Spain in 2011–2012 among individuals aged 18 years or over, the prevalence of obesity for men and women in Spain was 18.0% and 16.0%, respectively.[22] Because of this we decided to include BMI as a baseline variable of our cohort. The body mass index (BMI), calculated as the body mass divided by the square of the body height, was used to classify the cohort into underweight (<18.5 kg/m^2^), normal-weight (between 18.5 and 24.9 kg/m^2^), overweight (between 25 and 29.9 kg/m^2^) or obese (>30 kg/m^2^) categories. In a first step, we used an active approach of selecting healthy volunteers matched by gender, age, and years of education with a known cohort of patients with mTBI. However, the 1:1 matching process was not attained in some cases (for example, for young patients with low education levels) and eventually the selection criteria were limited to the ones already stated, regardless of the composition of the mTBI group. This study was reviewed and approved by the Ethics Committee of the Vall d´Hebron Research Institute (PR-AG-47-2013) and all participants signed a written informed consent.

### Assessment procedures

The present study focuses on the concussion standardized evaluation, carried out with the **SCAT2**.[15] The structure of SCAT2 is described in **Table 1**. In brief, in the symptom checklist the respondent evaluates the presence/absence of 22 post-concussion predefined symptoms and rates each symptom intensity on a Likert scale from 0 to 6. This results in two scores: the number of endorsed symptoms at evaluation and their severity. Items evaluating orientation, working memory and verbal memory are summed up in a cognitive index known as Standardized Assessment of Concussion (SAC) score, with a minimum value of 0 and a maximum of 30 points. For the purposes of this study we used as endpoints both the total number of endorsed symptoms (0–22) and their total severity score (0–132). The symptom profile was divided into four clusters: somatic, cognitive, emotional and fatigue-sleep domains (**Table 1)**. The probability of endorsing a symptom cluster is linked to the number of items it compasses (i.e. 9 for somatic versus 3 for sleep-fatigue). As the participants had not sustained any head trauma, the items regarding the postconcussive physical signs (loss of consciousness, balance difficulties) and the Glasgow Coma Scale were not applied and the maximum score for these components was granted in computing the SCAT2 total.

**Table 1:** The components of the Sport Concussion Assessment Test 2^nd^ edition (SCAT2). The following editions (SCAT3 and SCAT5) preserve this structure with minor scoring modifications and new supplementary material.

Self-report symptom check-list			
Somatic	Cognitive	Emotional	Fatigue/sleep
• Headache	• Feeling slowed down	• More emotional	• Trouble falling asleep
• ‘Pressure in head’	• Feeling like ‘in a fog’	• Irritability	• Drowsiness
• Neck pain	• ‘Don’t feel right’	• Sadness	• Fatigue / low energy
• Nausea or vomiting	• Difficulty concentrating	• Nervous or anxious	
• Dizziness	• Difficulty remembering		
• Blurred vision			
• Balance problems			
• Sensitivity to light			
• Sensitivity to noise			
**Cognitive examination *(Standardized Assessment of Concussion)***			
• *Immediate memory*: 3 trials of a list of 5 unrelated words			
• *Delayed memory*: free recall of the previously taught list			
• *Orientation*: 5 questions of time orientation			
• *Concentration*: backwards repetition of digit series and months of the year in reverse order			
**Other components**			
**Balance examination:** Modified Balance Error Scoring System (M-BESS)			
**Coordination:** finger-to-nose test			
**Physical signs:** loss of consciousness, balance difficulties			
**Glasgow Coma Scale:** standard neurologic evaluation			
**Maddocks Score:** optional—only suitable for sideline evaluation in sports-related concussion			

SCAT3: third version of Sport Concussion Assessment Test; SCAT5: fifth version of Sport Concussion Assessment Test

The **Hospital Anxiety and Depression Scale** (HADS) is a short self-report multiple-choice questionnaire, frequently used for screening clinically significant anxiety and depression in patients attending any general clinical setting.[23] The respondent is asked to fill in the answers in order to reflect how they have been feeling during the previous week. The anxiety and depression subscales are assessed separately. Each subscale has 7 items that can be graded from 0 to 3 points, therefore the scores range from 0 to a maximum of 21. The scores between 0 and 7 are classified as normal, between 8 and 10 as borderline, and 11 points or more as abnormal. The total HADS score ranges from 0 to 42, with higher values indicating more emotional distress. HADS’s screening properties are as good as other more comprehensive instruments used for identification of anxiety and depressive disorders.[24]

In addition, participants underwent a broader set of procedures that included detailed neuropsychological assessment and blood sampling for biomarkers and genetic determination (data not presented here). The study protocol also included performing a magnetic resonance imaging (MRI) within 1 year of the clinical evaluation, although generally it was done within 8 weeks (median 22 days, range 1–333 days). The MRI scanning was performed with a SIEMENS Magnetom TrioTim syngo 3-tesla equipment; data from a high-resolution 3D Magnetization Prepared Rapid Gradient Echo (MP-RAGE) protocol in addition to Fluid Attenuated Inversion Recovery (FLAIR) and echo gradient T2 sequences were assessed and informed by an expert neuroradiologist in all cases.

### Statistical analysis

Descriptive statistics were obtained for each variable. The Shapiro-Wilk test and inverse probability plot were used to test whether data followed a normal distribution. The mean and the standard deviation were used to describe continuous variables that followed a normal distribution and the median, maximum, and minimum values for continuous variables that were not normally distributed. Percentages and sample sizes were used to summarize categorical variables. To compare between-group differences (in categorical variables) χ2 statistics or the Fisher exact test were used as appropriate. Between-group differences were determined by an independent 2-sample *t*-test or the Mann–Whitney U test, depending on the statistical distribution. To correlate 2 continuous variables, the Kendall tau (when data did not follow a normal distribution) or Pearson correlation test (for data following a normal distribution) were used. The threshold for statistical significance was lowered from the routine *p* value of 0.05 and statistically significance was considered when *p* < 0.005.[25] This decision was taken following recent suggestions by many authors to change the default *p* -value threshold for statistical significance from 0.05 to 0.005, in particular in pilot studies, like ours, and with small sample sizes, as the risk of reporting false positive results is higher[25, 26].

To calculate the reference intervals (RIs) for the number of symptoms endorsed and the symptom severity score, the first step was to apply the Horn’s algorithm[27] to detect outliers. If Horn’s algorithm detected a significant outlier, the data was considered doubtful and eliminated of the RIs calculation. To calculate the upper and lower RI limits we used the distribution-free nonparametric method described in the NCCLS and Clinical and Laboratory Standards Institute (CLSI) guidelines C28-A3 for estimating percentiles intervals[28, 29] by using the package ‘referenceIntervals’ for R.[30]

Statistical analyses were carried out with Microsoft-enhanced R distribution (Microsoft R Open 3.4.4, Microsoft corporation 2017, https://mran.microsoft.com) and the integrated development environment R Studio v1.1.453 (RStudio, Inc., Boston, MA, USA; http://www.rstudio.com). The following R packages were used in the analysis: XLConnect 0.2.13, Hmisc 4.0.3, referenceIntervals 1.1.1 and car 2.1.6.

## Results

### Participants

The group of healthy volunteers consisted of 60 participants, out of which 38 (63.3%) were men, whose socio-demographic characteristics are summarized in **Table 2.** The sample had the following distribution in terms of age groups: 26.7% between 18–24, 28.3% between 25–34, 16.7% between 35–44, 11.7% between 45–54 and 16.7% between 55–64 years old. Of the entire cohort, 49 cases (81%) achieved at least 12 years of education. Based on the BMI values, 69.5% of participants were normal-weight and 23.7% were categorized as overweight.

**Table 2:** Socio-demographic characteristics of the cohort (n = 60).

**Sex** (men/women: n, %)	38 (63.3) / 22 (36.7)
**Age** (years: mean ± SD, min, max)	36.2 ± 13.9 (18–64)
**Education level** (years: mean ± SD, min, max)	13.8 ± 3.6 (8–22)
**Education level** (higher level achieved)	
Primary education (n)	9
Secondary education (n)	2
High-school education or professional training (n)	23
Bachelor studies (n)	12
Postgraduate studies (n)	14
**Body Mass Index**^1^ (kg/m^2^: mean ± SD, min, max) (n = 59)	23.51 ± 2.97 (17.35–31.14)
Underweight (n)	4
Normal weight (n)	41
Overweight (n)	13
Obese (n)	1
**HADS anxiety**^1^ (median, min, max) (n = 59)	5 (1–14)
Normal (n)	45
Borderline (n)	8
Elevated (n)	6
**HADS depression**^1^ (median, min, max) (n = 59)	2 (0–8)
Normal (n)	57
Borderline (n)	2
Elevated (n)	0

HADS: Hospital Anxiety and Depression Scale; max: maximum score; min: minimum score.

^1^ the BMI and the HADS scores are available for 59 participants.

### Neuroimaging findings

**Fig 1:**
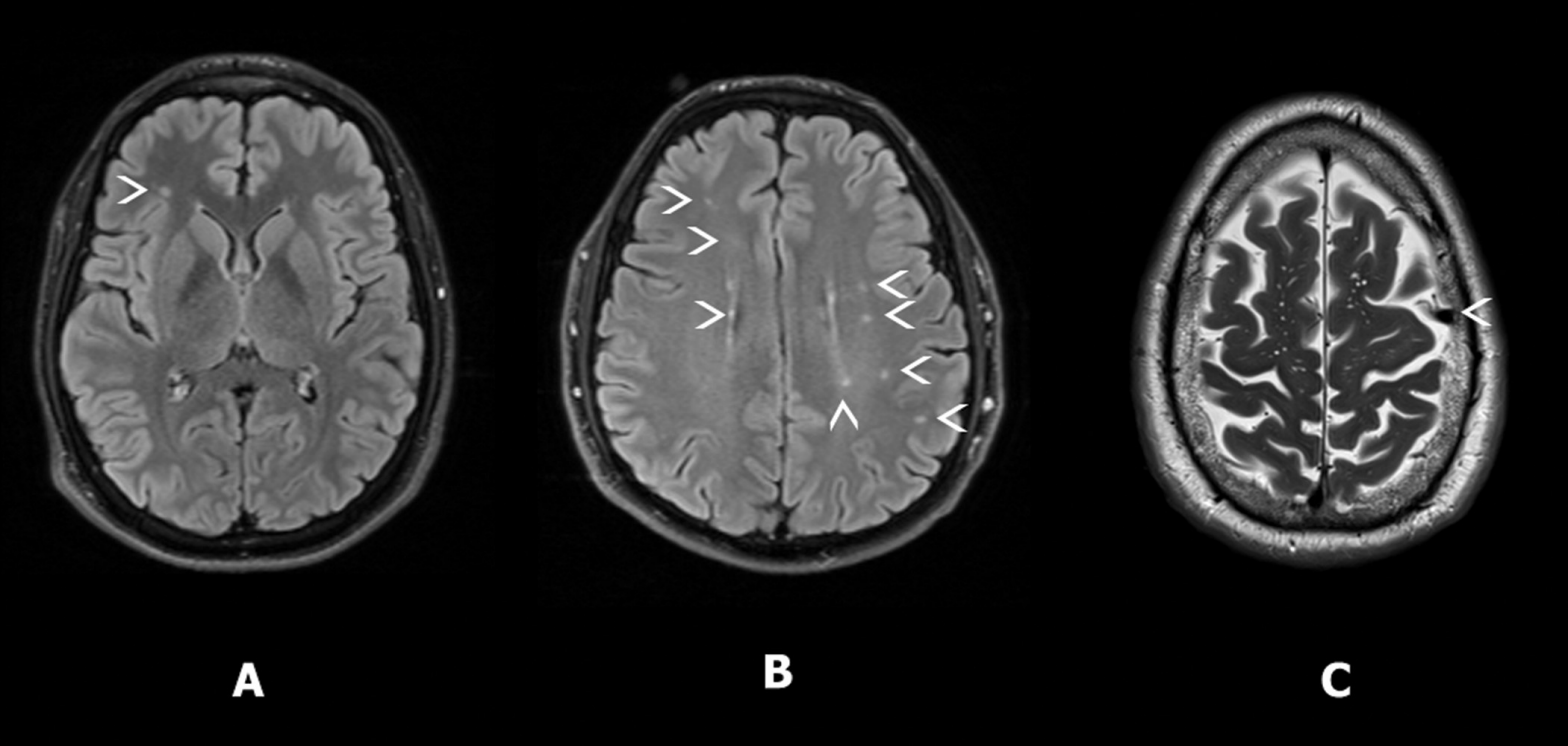
Examples of MRI incidental findings. Relevant details are marked with white arrowheads: A. Unspecific punctate lesion in a 21-year old male, in FLAIR. B. Multiple foci of white matter hyperintensity, corresponding to stage 1 on the Fazekas scale,[32] visible in the FLAIR scan in a 50-year old man. C. Venous angioma in a 58-year old man in a T2-weighted image.

The MRI findings for 56 volunteers are summarized in **Table 3**. The neuroradiological information was not available in 4 of the 60 cases: in one case due to technical problems, in a second because of claustrophobia, in a third out of safety concerns for a volunteer with an implant of undocumented material and in the fourth because the patient refused the MRI. Forty participants (71.4%) did not show any abnormality. Incidental findings of weak or no clinical relevance were reported in 16 cases (28.6%). Most of these were unspecific foci of T2/FLAIR signal abnormality (**Fig 1**). The Evans’ Index (EI) represents a rough indicator of ventricular volume and was also computed. The EI is the ratio between the maximum width between the anterior horns of the lateral ventricles and the maximal internal diameter of skull, measured at the same level.[31] An EI value that exceeds 0.30 warrants further examination, and in this sample was 0.24 on average and varied between 0.20 and 0.29. All cases were thoroughly revised and lacked any medical condition that required excluding them from the group.

**Table 3:** Magnetic Resonance Imaging (MRI) findings (n = 56).

Nothing remarkable	40
Unspecific foci of T2/FLAIR signal abnormality	7
Punctiform white matter lesions (Fazekas 1)	3
Small venous angioma	3
Mild diffuse or focal atrophy	2
Microbleeding (possible cavernoma)	1

T2/FLAIR: T2-weighted or fluid attenuated inversion recovery (FLAIR) sequence

### SCAT2 assessment and the reference interval calculation

**Fig 2:**
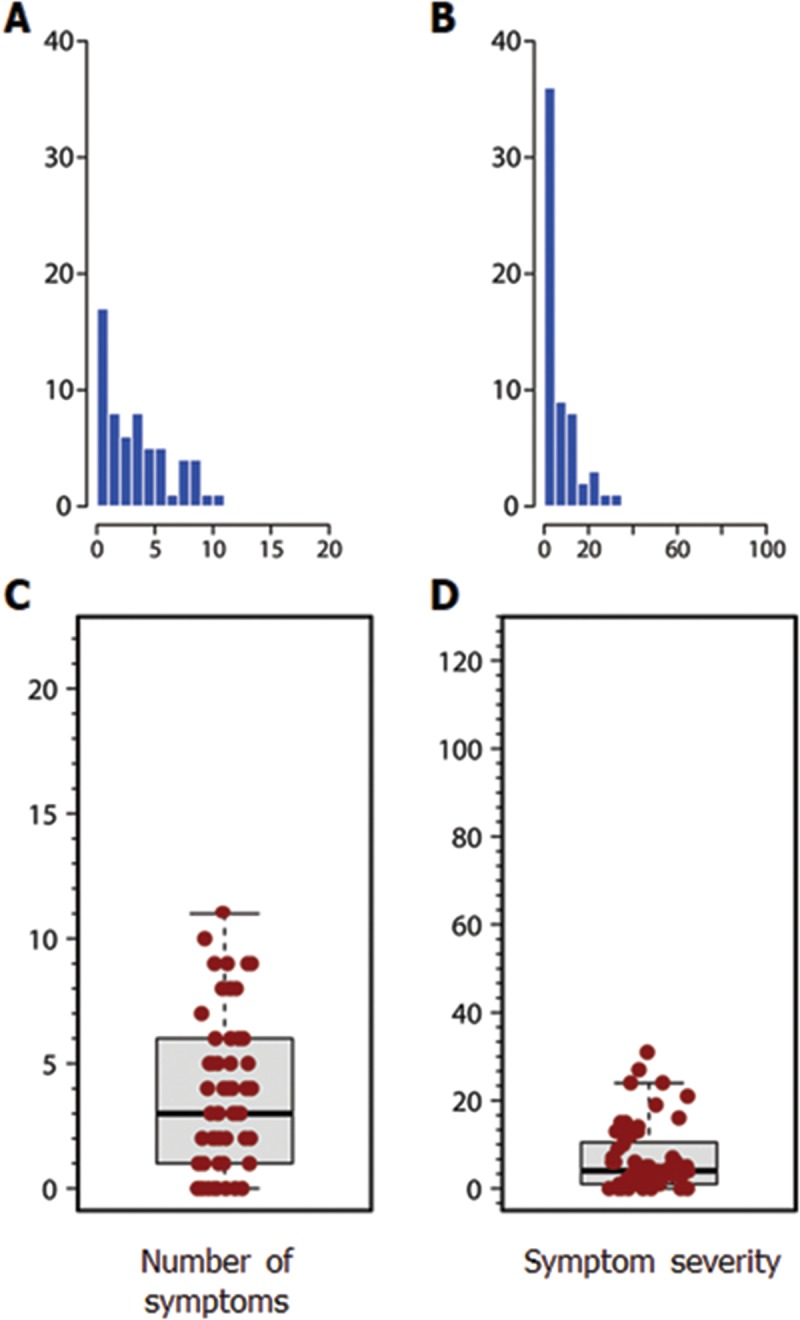
Distribution of the number of symptoms and the symptom severity score in the cohort. A. Histogram for the number of symptoms. B. Histogram for the symptom severity score C. Box-and-whiskers plot for the number of symptoms. D. Box-and-whiskers plot for the symptom severity score. The black lines inside the boxes are the median values for each group. The vertical size of the boxes is the interquartile range (IQR). The ‘whiskers’ represent the minimum and maximum values that do not exceed 1.5×IQR.

The symptom profile, as reported by the volunteers, and the scores for all SCAT2 components are presented in **Table 4**. The participants reported a median of 3 symptoms (min-max: 0–11) **(Fig 2C**), with a severity score of 4 (min-max: 0–31) (**Fig 2D**). Encountering difficulties in the balance examination was frequent, as the M-BESS score had a median of 24, out of a maximum of 30 points. In the cognitive evaluation, the concentration and delayed memory scores varied the most, regardless of a high total SAC score (median: 27; min-max: 22–30). Orientation and immediate memory scores were perfect or nearly perfect in all cases.

**Table 4:** SCAT2 scores and postconcussive-like symptom profile.

	**median (min—max)**
**Number of symptoms**	3 (0–11)
**Severity score**	4 (0–31)
**Balance BESS**	24 (14–30)
**SAC scores**	27 (22–30)
• Orientation	5 (4–5)
• Concentration	4 (2–5)
• Memory immediate	15 (13–15)
• Memory delayed	4 (0–5)
**SCAT2 total**	87 (71–97)
**Most frequently reported symptoms**	**n (%)**
• Difficulty concentrating	25 (41.7)
• Difficulty remembering	21 (35.0)
• Fatigue or low energy	21 (35.0)
• Nervous or Anxious	20 (33.3)
• Drowsiness	16 (26.7)
• Sadness	13 (21.7)
• Neck pain	12 (20.0)
• Trouble falling asleep	12 (20.0)
• Headache	11 (18.3)
**Symptom clusters**	
• Somatic	33 (55.0)
• Cognitive	34 (56.7)
• Emotional	26 (43.3)
• Sleep—fatigue	33 (55.0)

SCAT2: Sport Concussion Assessment Test, 2nd edition; BESS: Balance Error Scoring System; SAC: Standardized Assessment of Concussion

The most frequently endorsed symptom was feeling difficulty in concentrating, with 41.7% of the sample reporting it on the day of the examination. In addition, more than 1 in 3 participants reported feeling difficulty remembering, fatigue or low energy and feeling nervous or anxious. Sixteen volunteers (26.7%) endorsed more than 5 symptoms. Fifteen out of the 22 symptoms were endorsed by at least 10 percent of the sample. The exploration of the symptom profile at cluster level also indicated heterogeneity. Participants reported symptoms in the somatic, cognitive or sleep-fatigue clusters with similar frequencies (55%, 56.7% and 55% respectively), while 43.3% of the sample endorsed at least one emotional symptom.

The number of symptoms and the severity score distributions were significantly skewed to the right (**Fig 2A and 2B**) (Shapiro-Wilk’s test for symptom number: W = 0.92, *p* = 0.001 and for the severity score: W = 0.82, *p* <0.001). For both scores, the Horn’s algorithm did not flag any outlier. Therefore, the entire cohort was included in the calculation of the RIs. As previously described, the distribution-free nonparametric reference intervals method was used, that calculates the 2.5 and the 97.5 percentiles. The upper interval boundary obtained was 10.5 for the symptom number, and 28.9 for the severity score.

### Screening anxiety and depression

The HADS scores are presented in **Table 2**. Nearly the entire sample (96.7%) scored less than 8 points on the depression subscale, while only 2 cases were classified as borderline and no participant reported symptomatology that could have been considered clinically relevant. Although anxiety symptoms were more prevalent, 76.3% of participants scored in the normal range. Another 13.6% were borderline and the remaining 10% scored above the threshold indicative of a potential anxiety disorder.

### Factors related to concussion-like symptom profile

In analyzing whether the symptom presentation differs by sex, women endorsed a median of 3.5 symptoms (min: 0, max: 9) and men a median of 3 symptoms (min:0, max: 11). These differences were not statistically significant (Mann-Whitney U Test, W = 404, p = 0.83). The median severity score for men was 4 (min: 0, max: 31) and 5 for women (min: 0, max: 27). The differences in severity score were also not statistically significant (Mann-Whitney U Test, W = 415.5, p = 0.97).

To test if endorsed symptoms and their severity increased with age, Kendall's rank correlations were performed. The results showed no statistically significant association between age and endorsed symptoms (Τ = 0.14, *p* = 0.13), nor between age and the severity score (T = 0.16, *p* = 0.07).

Scatterplots were constructed to correlate the HADS total score, the anxiety subscale (HADS-A) and the depression subscale (HADS-D) with the number of endorsed symptoms and the severity score. At the predefined alfa level of 0.005, we did not find any association between the HADS total score and the endorsed symptoms (Τ = 0.19, *p* = 0.037) or the HADS total score and the severity score (Τ = 0.22, *p* = 0.019). We did not find any correlation when plotting the HADS-A score against the number of the endorsed symptoms (Τ = 0.18, *p* = 0.068) or the HADS-A score against the severity score (Τ = 0.21, *p* = 0.029). No association was found between HADS-D and the number of the endorsed symptoms (Τ = 0.19, *p* = 0.057) or the severity score (Τ = 0.18, *p* = 0.067).

## Discussion

The incidence of post-concussion syndrome is widely heterogeneous, it ranges between 10–30% among different patient populations, and the diagnostic criteria are still inconsistent.[33, 34] Even the minimum number of symptoms and the time since injury required for diagnosis are a matter of debate, with the latter varying in different studies from 7 days to 3 months.[33, 35, 36] In the clinical evaluation and research studies of non-sport mTBI, inventories of self-reported symptoms are frequently used. The SCAT2 is the most widely-used structured tool in sports-related concussion assessment and it includes a 22-item self-report symptom scale.[15] Athletes usually have a preseason SCAT2 evaluation baseline, but in the clinical setting baseline measurements are lacking and therefore clinicians cannot perform the comparison of endorsed symptoms and their severity. It has been shown that ‘healthy’ individuals in the general population frequently report concussion-like symptoms. In order to identify symptomatology that manifests in the absence of head injury, most previous studies have targeted sports-related concussion and have described baseline evaluations of youth or collegiate athletes.[12, 21, 37] Some self-report tools, like the Rivermead Post-concussion Questionnaire,[38] tackle the absence of baseline evaluations by asking the respondent to distinguish between symptoms that have been present beforehand and others that have only become a problem following injury. Although this is a valuable approach, its reliability is limited by the ‘good old days’ bias, as patients with mTBI retrospectively report their preinjury status as better than the average person.[39] The aim of this study was to establish a population-based threshold for SCAT2 symptom profile indicators—number of symptoms, clusters and severity score—that could be used in multivariate analysis of non-sports mTBI cohorts with the purpose of discriminating patients with risk factors for presenting clinically-relevant PPCS that significantly affect patients' psychosocial functioning and of identifying different biomarkers that could predict them.

This study reports the reference intervals for concussion-like symptoms in a ‘healthy’ adult population between 18 and 65 years of age. Our data showed that non-concussed individuals frequently reported concussion-like symptoms. In this cohort, uninjured participants reported a median of 3 concussion-like symptoms and the upper reference interval was found at 10.5 symptoms, out of a total of 22. The median severity score was 4.9 points and 28.9 was the upper limit for the reference interval. It is worth noting that only 10 participants (16.7%) did not endorse any symptom. This is in agreement with the findings of Iverson et al. who showed in high school athletes that 19% of boys and 28% of girls reported having a symptom burden resembling the diagnosis of post-concussion syndrome.[37]

Clinical research needs consensus in identifying goals that are clinically relevant and, in mTBI outcome assessment, this calls for agreement on a minimum set of symptoms and/or severity score. However, this is not yet the case. A recent survey on physician members of the American Society of Sports Medicine showed that 55% of the respondents considered that just ‘1 symptom’ was enough for the diagnosis of post-concussion syndrome, while 17.6% of the participants required at least two symptoms.[33] The fact that our study yielded such elevated cut-offs scores in a healthy population makes low threshold used in sport injuries misleading when applied to a civilian population and warrants careful interpretation of the results of the SCAT2 inventory or any similar self-reported symptoms checklist in non-sports settings. In addition, establishing the clinical value of symptoms entails determining whether patients’ symptoms are a consequence of the concussion or of other factors, especially when symptoms are reported weeks or months after injury. In this complex decision-making process, a criterion of any arbitrary number of symptoms that is to be used independently of their severity can only add to the confusion. We addressed this by conducting a population-based approach with a thorough characterization of participants that included MRI scanning and by using robust methods to flag outliers and establish an upper reference interval for self-reported symptoms. In addition to the clinical relevance of the present data, supervised machine learning models of mTBI could benefit from incorporating data-driven outcome thresholds. Supervised learning strategies—i.e. that aim to predict predefined output values from several input measures, such as logistic regression or random forests,[40] need to take into account the limitations of the traditional mTBI outcome scores, when symptoms commonly found in the general population are not considered.

It is worth mentioning that the MRI incidental findings in our cohort were not unexpected for a healthy sample, taking into account the high detection capability of 3 Tesla machines.[41] White matter hyperintensities (WMHI) were the most frequent finding in our sample (12.5%). In a cohort in which 41% of the participants were between 40 and 65 years old, the results are not remarkable. [42] As explained, the decision to not exclude participants with incidental findings from our study was made after careful considerations of their clinical background and known health status. In addition, other studies have called upon not excluding participants with WHMI from mTBI control groups[43], especially in DTI research. The exclusion of cases with common preinjury characteristics, like WHMI, that would not be excluded from a mTBI sample has potentially resulted in a systematic bias. In the present study, we have opted for a sample with increased representativity for the general adult population.

Looking further into the SCAT2 results, one factor that could explain the high incidence of concussion-like symptoms in this cohort is the method of assessment. As previously stated, the SCAT2 was chosen for its wide use in sport-related concussion literature and its suitability for clinical practice. However, various studies have shown that there is a statistically significant difference in symptom reporting when using different tools with open-ended questions, a simulated structured interview or a standardized checklist. The standardized checklists like the SCAT2 are the form of assessment that elicits the most symptoms, both in non-concussed students [44] and in patients with mTBI.[45] As previously stated, this highlights the need of consensus in mTBI medicine and that clinicians evaluating patients with mTBI need to be cautious when interpreting self-reported questionnaires.

Previous literature has tended to show that women endorse more symptoms than men, either as control volunteers or as athletes in preseason baseline evaluation and following concussion, and even that they are more likely to report different symptoms than men.[21] However, in this cohort, there was no effect of gender and age on symptom presentation. Whether or not this is a robust pattern should be addressed by replication studies with a bigger sample size. It is also possible that the general population controls have different profiles than young college students and high-school athletes and, therefore, the observed differences in other studies could reflect the distinct composition of the studied samples.

Emotional distress is a frequent reaction to a traumatic event and it plays an important role during the recovery process. In mTBI research and in PPCS assessment, depression has always been considered a pivotal factor as it can trigger or exacerbate the manifestation of other post-concussion symptoms. Specifically, depressive and anxiety symptomatology is associated with fatigue, low energy and trouble sleeping and it is a better predictor for presenting cognitive complaints than objective neuropsychological functioning.[46] In this cohort, the levels of depression and anxiety were not linked to concussion-like symptom presentation. These results were produced by applying a stringent threshold for statistically significance (p < 0.005), arguably failing to report weak correlations between emotional distress and both symptom number and their severity, and between anxiety and symptom severity (Tau between 0.19 and 0.22, p < 0.05, Kendall’s). Regarding depression, the results are explained by the absence of participants with evident depressive symptomatology. As few moderate and no high scores were obtained in the HADS-D subscale, the variation in the profile of concussion-like symptoms could not be linked to depression.

### Study limitations

Several limitations to this study are acknowledged. The participants were selected from a single tertiary hospital and this may limit generalizability. In addition, the enrolment of next-of-kin of patients admitted to a neurosurgical department could result in a cohort with higher stress levels than community population. All preexisting health conditions were self-reported and were not verified. In addition, because participants were selected with predefined demographic characteristics, this cohort was not representative of the Spanish population in terms of age and gender distribution. It disproportionately includes more men, and more participants between 18–24 years of age and less participants between 25–54. Furthermore, the sample size (n = 60) is relatively small, in comparison with other multi-center studies that present normative data for concussion assessment. Future studies should be directed at improving normative data in specific subgroups of civilian population by increasing the sample size and including elders. As explained previously, clinical anxiety and depression symptoms can modulate the presentation and resolution of mTBI symptomatology. Regardless general anxiety or depression levels, because our cohort includes companions and relatives of patients, it is possible that they pay more attention to their own body and mental state and could even exhibit a different pattern of symptoms, as biased by this exposure to health issues. Future studies could address how concerns about one’s health (in relation with specific stressors like the illness of a relative) relate to the manifestation of symptoms in the general healthy population.

In addition, falls in people over 70 years old are the most frequent scenario of mTBI, but the particularities of injury mechanism, comorbidities and preinjury treatment in this cohort complicate its inclusion in a general adult mTBI sample. Therefore, assessing the necessity of establishing different normative scores for these distinct mTBI populations would be a valuable advancement of the present study.

### Conclusions

PPCS are a cause on ongoing disability and distress for affected patients and a source of high healthcare costs. Further studies directed toward identifying individuals who are at risk for developing PPCS after mTBI are important to both patients and healthcare professionals. Prognosis models should not rely on the use of arbitrary cut-off scores for symptom-related variables, because they currently fail to reflect the frequent presentation of similar symptoms in the absence of prior head injury. Our results suggest that for a better refinement of the patient selection, the outcome variables should be redefined to take into consideration that some concussion-like symptoms can be endorsed by healthy individuals.

## Abbreviations

ADHD: attention-deficit/hyperactivity disorder
BMI: body mass index
CT: computed tomography
DTI: diffusion tensor imaging
ED: emergency department
EI: Evans index
FLAIR: fluid-attenuation inversion recovery
GCS: Glasgow Coma Scale
HADS: Hospital Anxiety and Depression Scale
LOC: loss of consciousness
M-BESS: modified Balance Error Scoring System
MP-RAGE: magnetization-prepared rapid gradient-echo
MRI: magnetic resonance imaging
mTBI: mild traumatic brain injury
PPCS: persistent post-concussion symptoms
PTA: posttraumatic amnesia
SAC: Standardized Assessment of Concussion
SCAT2: Sport Concussion Assessment Tool 2^nd^ Edition
TBI: traumatic brain injury
WHO: World Health Organization
